# Cumulative lactate and hospital mortality in ICU patients

**DOI:** 10.1186/2110-5820-3-6

**Published:** 2013-02-27

**Authors:** Paul A van Beest, Lukas Brander, Sebastiaan PA Jansen, Johannes H Rommes, Michaël A Kuiper, Peter E Spronk

**Affiliations:** 1 Department of Anaesthesiology, University Medical Center Groningen, University of Groningen, PO Box 30001, Groningen 9700 RB, the Netherlands; 2 Department of Intensive Care Medicine, Medical Center Leeuwarden, PO Box 888, Leeuwarden, 8901 BR, the Netherlands; 3 Department of Intensive Care Medicine, Gelre Hospitals, location Lucas, PO Box 9014, Apeldoorn, 7300 DS, the Netherlands; 4 Department of Anesthesia and Intensive Care Medicine, Luzerner Kantonsspital, Luzern, 6000, Switzerland; 5 HERMES Critical Care Group, Amsterdam, The Netherlands

**Keywords:** Lactate, Critically ill, Intensive care units, In-hospital mortality

## Abstract

**Background:**

Both hyperlactatemia and persistence of hyperlactatemia have been associated with bad outcome. We compared lactate and lactate-derived variables in outcome prediction.

**Methods:**

Retrospective observational study. Case records from 2,251 consecutive intensive care unit (ICU) patients admitted between 2001 and 2007 were analyzed. Baseline characteristics, all lactate measurements, and in-hospital mortality were recorded. The time integral of arterial blood lactate levels above the upper normal threshold of 2.2 mmol/L (lactate-time-integral), maximum lactate (max-lactate), and time-to-first-normalization were calculated. Survivors and nonsurvivors were compared and receiver operating characteristic (ROC) analysis were applied.

**Results:**

A total of 20,755 lactate measurements were analyzed. Data are srpehown as median [interquartile range]. In nonsurvivors (n = 405) lactate-time-integral (192 [0–1881] min·mmol/L) and time-to-first normalization (44.0 [0–427] min) were higher than in hospital survivors (n = 1846; 0 [0–134] min·mmol/L and 0 [0–75] min, respectively; all *p* < 0.001). Normalization of lactate <6 hours after ICU admission revealed better survival compared with normalization of lactate >6 hours (mortality 16.6% vs. 24.4%; *p* < 0.001). AUC of ROC curves to predict in-hospital mortality was the largest for max-lactate, whereas it was not different among all other lactate derived variables (all *p* > 0.05). The area under the ROC curves for admission lactate and lactate-time-integral was not different (*p* = 0.36).

**Conclusions:**

Hyperlactatemia is associated with in-hospital mortality in a heterogeneous ICU population. In our patients, lactate peak values predicted in-hospital mortality equally well as lactate-time-integral of arterial blood lactate levels above the upper normal threshold.

## Background

Hyperlactatemia is common in critically ill patients and may reflect an imbalance between local or systemic oxygen supply (DO_2_) and oxygen consumption (VO_2_). Hyperlactatemia also may be found during increased aerobic glycolysis in hypermetabolic states from various causes [1,2], in patients treated with catecholamines [3,4], as a consequence of alkalosis in hyperventilation [5], and with impaired hepatic lactate clearance in sepsis or low flow states [6]. Elevated lactate levels are associated with the development of multiple organ dysfunction (MODS) postoperatively, following trauma, and septic shock [7-10], and it has been suggested that hyperlactatemia is associated with worse outcome [10-13]. Persistence of lactate levels above normal is associated with higher mortality rates in patients with severe sepsis, septic shock [9,14], and in postcardiac arrest patients [15].

We hypothesized that the severity of persistent hyperlactatemia represented by the time integral of arterial blood lactate levels above the upper normal threshold of 2.2 mmol/L (lactate-time-integral) outperforms single lactate measurements in predicting outcome. We therefore retrospectively investigated the relationship between lactate derived variables (admission level, maximum level, time-to-first-normalization, lactate-time-integral) and in-hospital mortality in a large, mixed intensive care unit (ICU) population. Subgroup analysis was performed on categories in which lactate has been described as predictor of mortality (sepsis and circulatory failure) [10-13]. Additionally, we looked at possible differences between survivors and nonsurvivors in the 24 hours after admittance [16].

## Methods

### Setting

A retrospective, observational study in a university-affiliated teaching hospital where the ICU is a mixed, ten-bed, “closed format” department. There were no changes in medical staff during the study period. Case records from all ICU patients with available lactate measurements admitted during a 5-year period, January 2002 to December 2006, were identified in the ICU database. The study was approved by the Local Ethics Committee, which waived the need for informed consent.

### Data collection

Data from all days spent in the ICU were collected retrospectively from the electronic patient data monitoring system and the hospital administration database. We collected demographic information, diagnosis, acute physiology and chronic health evaluation (APACHE II), all lactate levels, and relevant variables for calculation of daily assessed SOFA score (Table 1). Diagnosis classifications were based on the APACHE II classifications, hence diagnosis category weight [17]. Finally, length of stay in the ICU (LOS_ICU_), days in the hospital before discharge (LOS_HOSP_), and hospital survival were recorded.

**Table 1:** Baseline and clinical characteristics

**Characteristic**	**All**	**Survivors**	**Nonsurvivors**	***p *value**^**a**^
	**n = 2,251**	**n = 1846**	**n = 405**	
Age (yr)	66 (12–98)	69 (57–76)	75 (67–81)	
Sex M : F (%)	61 : 39	61 : 39	60 : 40	
SAPS-II	38 (20–113)	33 (24–43)	51 (41–65)	<0.001^*^
SOFA				
APACHE II	17 (10–54)	15 (11–19)	23 (17–28)	<0.001^*^
Admission source (%)				<0.01^#*^
Emergency	20.2	20.4	19.3	
Surgical / OR	43.7	47.3	27.0	
Medical	27.2	23.7	43.1	
CCU	8.0	7.5	10.1	
Other	0.9	1.1	0.5	
Diagnosis (%)				<0.01^#*^
Vascular surgery	16.0	17.2	10.4	
Abdominal surgery	22.4	23.5	17.2	
Other surgery	9.8	10.9	4.9	
Heart failure	14.8	12.3	26.4	
Respiratory failure	11.8	11.5	13.4	
GI bleeding	3.8	4.3	1.5	
Neurological	4.5	4.6	2.7	
Other	3.7	3.9	4.2	
Sepsis	13.1	11.8	19.2	
Vasoactive agent (%)	33	28	57	<0.001^*^
LOS ICU (days)	2 (1–5)	2 (1–5)	3 (1–8)	<0.001^*^
LOS HOSP (days)	14 (7–27)	15 (9–28)	6 (2–16)	<0.001^*^
In-hospital mortality (%)	18			

Data are presented as numbers and median (interquartile range). AUC, area under the curve; SAPS-II, simplified acute physiology score; APACHE II, acute physiology, age and chronic health evaluation; OR, operating room; CCU, cardiac care unit; GI, gastrointestinal; vasoactive agent: noradrenaline, dopamine, dobutamine, phosphodiesterase inhibitor; LOS ICU, length of stay at intensive care; LOS HOSP, length of stay at hospital; ^a^Survivors vs. nonsurvivors; ^b^All normal lactate (AUC = 0) vs. all elevated lactate (AUC > 0); ^*^Statistically significant difference. Statistics by Chi-square tests^#^ and Wilcoxon rank-sum tests.

### Lactate levels and derived variables

**Figure 1:**
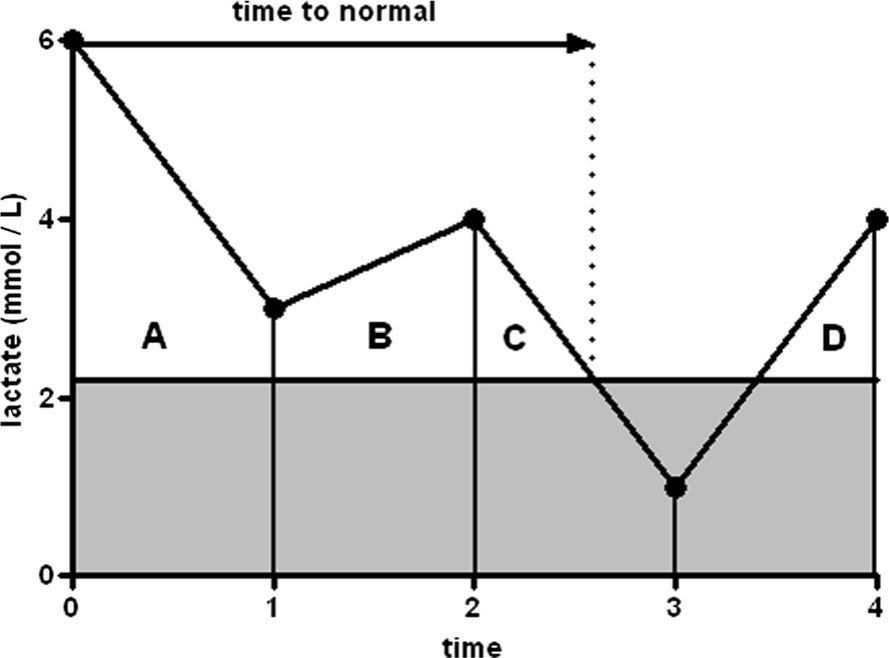
**Calculation of lactate area under the curve above the upper normal limit of lactate (2.2 mmol/L). **The following four equations were used in computing lactate-AUC: 1) AUC A = ((½· (lactate 0 – lactate 1)) + lactate 1)) · (time 1 – time 0); 2) AUC B = ((½· (lactate 2 – lactate 1)) + lactate 1)) · (time 2 – time 1); 3) AUC C = ((lactate 2 – 2.2)^2^ · (time 3 – time 2)) / 2 · lactate 2; 4) AUC D = ((lactate 4 – 2.2)^2 ^· (time 4 – time 3)) / 2 · lactate 4.

Lactate levels were measured in arterial blood using point-of-care blood gas analyzers (Rapidlab 865, Siemens, Munich, Germany; upper normal limit 2.2 mmol/L). The time integral for lactate levels above the upper normal threshold of 2.2 mmol/L was calculated during the entire ICU stay (lactate-time-integral) using custom-made software. We used a formula that, for practical reasons, assumed a linear change over time between measurements. Figure 1 illustrates four possible scenarios used for calculating lactate-time-integral.

Lactate buffer solutions for renal replacement therapy (RRT) and continuous epinephrine infusion were not used during the study period following the general policy in the unit.

### Statistical analysis

The statistical package for the social sciences (SPSS 16.0.1 for Windows, Chicago, IL) was used for statistical analyses and additional software was used for graphics (Prism 5.0 for windows, La Jolla, CA) and comparison of ROC curves (MedCalc 11.2.1, Mariakerke, Belgium). Data are presented as mean ± SD or median [interquartile range] as indicated by assessment of normal distribution (D’Agostino-Pearson omnibus normality test). Mann–Whitney *U* test was used for not normally distributed data. Differences of admission source or admission diagnosis between groups of survivors and nonsurvivors and those with and without hyperlactatemia were assessed using the Chi-square test. Receiver operating characteristic (ROC) curves were used for the assessment of sensitivity and specificity of lactate-derived variables to predict in-hospital mortality. Areas under the ROC curves (AUC_ROC_) were compared by the method described by DeLong et al. [18]. Statistical significance was assumed at *p* < 0.05.

## **Results**

During the 5-year period, case records of 2,251 patients (age 66 [12–98] years; 39% female) were identified. From all patients, at least one lactate sample was drawn and therefore none of the patients was excluded. A total of 20,755 lactate measurements were analyzed. Median lactate samples per day per patient was 2.0 [1.0-5.0] samples. Median lactate concentration at admission was 1.7 [1.1-2.8] mmol/L; minimum 0.6 mmol/L and maximum 27.0 mmol/L. Median max-lactate was 2.1 [1.5-3.3] mmol/L, and median lactate-time-integral 0.0 [0.0-244] min·mmol/L. Baseline and clinical characteristics of all patients are summarized in Table 1.

**Figure 2:**
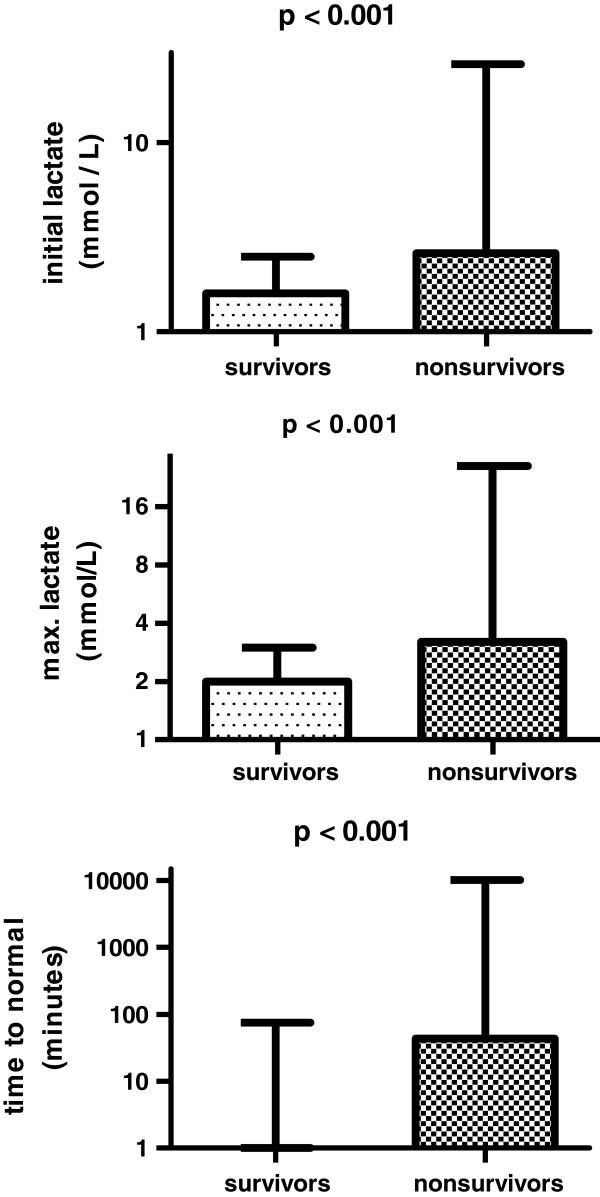
**Admission lactate levels (mmol/L), maximum lactate levels (mmol/L), time-to-normal (min) for survivors (n = 1.846) and nonsurvivors (n = 405). **Bars show median (upper interquartile range); Mann–Whitney test; logarithmic scale.

In-hospital mortality of our population was 18% and was higher in patients with hyperlactatemia during ICU stay compared with those without hyperlactatemia (26.4% vs. 10.8%; *p* < 0.001). Survival was significantly higher in patients with lactate normalization within 6 hours after ICU admission (n = 1,856) compared with patients with lactate normalization >6 hours after ICU admission (n = 395; 16.6% vs. 24.4%; *p* < 0.001). For patients who died in the hospital (n = 405), admission lactate (2.6 [1.5-5.0] mmol/L), max-lactate (3.2 [1.9-5.8] mmol/L) time-to-first-normalization (44.0 [0–427] min), and lactate-time-integral (192 [0–1,881] min·mmol/L), were higher than in-hospital survivors (n = 1,846; admission lactate (1.6 [1.1-2.5] mmol/L), max-lactate (2.0 [1.4-3.0] mmol/L), time-to-first-normalization (0.0 [0–75] min) and lactate-time-integral 0 [0–134] min·mmol/L, respectively; all *p* < 0.001; Figure 2). Subanalysis for the first 24 hours showed similar results; all *p* < 0.001.

**Figure 3:**
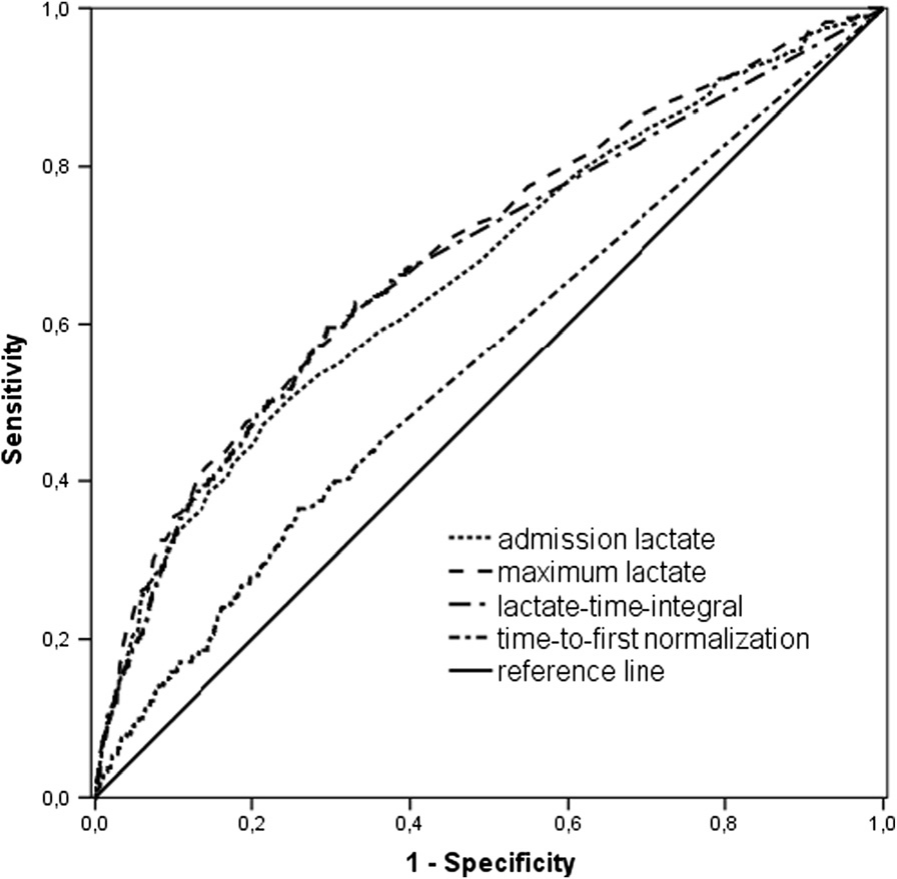
**Receiver operating characteristic curves for in-hospital mortality prediction. **Area under the curve was 0.552 for time-to-first-normalization, 0.666 for admission lactate, 0.676 for lactate-time-integral and 0.692 maximum lactate.

Figure 3 demonstrates the difference between admission lactate levels and lactate-derived variables with respect to predicting in-hospital mortality. AUC_ROC_ for admission lactate and lactate-time-integral were similar (0.666 [95% confidence interval (CI) 0.646-0.686] vs. 0.676 [95% CI 0.657-0.696]; *p* = 0.36). AUC_ROC_ for max-lactate (0.692 [95% CI 0.672-0.711]) was larger than AUC_ROC_ for admission lactate (0.666 [95% CI 0.646-0.686]; *p* = 0.01) lactate-time-integral (0.676 [95% CI 0.657-0.696]; *p* < 0.01), and time to normal (0.552 [95% CI 0.531-0.573]; *p* < 0.001). ROC curves for all four variables were significantly different from the reference line (all *p* < 0.01). The cutoff points derived from the ROC curve for admission lactate, max-lactate and cumulative lactate were 2.7 mmol/L, 2.5 mmol/L, and 53 min·mmol/L, respectively. AUC_ROC_ lactate-time-integral per day was (0.672 [95% CI 0.641-0.694]; figure not shown).

AUC_ROC_ for both time to normal and lactate-time-integral were also calculated with other thresholds (thresholds 1.0 mmol/L, 2.0 mmol/L, 3.0 mmol/L, and 4.0 mmol/L respectively). Table 2 summarizes the results for both total population and subgroups. None of these AUC_ROC_ were significantly different from the reference line (all *p* > 0.05).

**Table 2:** AUC ROC curves with various thresholds

**Threshold**^**a**^	**Lactate-time-integral**	**Time-to-first-normalization**^**b**^
Population		
1	0.504	0.514
2	0.504	0.514
3	0.513	0.518
4	0.504	0.507
Sepsis		
1	0.555	0.535
2	0.555	0.535
3	0.539	0.544
4	0.507	0.512
Cardiac failure		
1	0.524	0.509
2	0.524	0.509
3	0.486	0.493
4	0.493	0.482

AUC ROC, area under receiver operating characteristic curve; ^a^lactate threshold presented in mmol/L; ^b^normalization to respective threshold.

In none of the subgroups (sepsis, n = 307; cardiac failure, n = 213; Table 3) AUC_ROC_ for lactate-time-integral was larger than AUC_ROC_ for single lactate values. In both subgroups admission lactate, maximum lactate and lactate-time-integral differed between survivors and nonsurvivors (all *p* < 0.001). Analysis for the first 24 hours after admission showed similar results; in both subgroups all *p* < 0.01.

**Table 3:** Baseline and clinical characteristics subgroups

**Characteristic**	**Sepsis (n = 307)**	**Cardiac failure (n = 213)**
Age (yr)	69 [60–77]	72 [63–78]
Sex M : F (%)	63 : 37	60 : 40
SAPS II	48 [37–57]	48 [37–63]
APACHE II	21 [17-26]	22 [16–28]
Heart rate (beats/min)	115 [96–130]	110 [80–125]
Systolic blood pressure (mmHg)	105 [80–130]	110 [85–140]
Mean arterial pressure (mmHg)	58 [50–62]	59 [51–65]
Vasoactive agent (%)	56	56
Lactate (mmol/L)	2.4 [1.6-3.9]	2.4 [1.4-4.8]
Maximum lactate (mmol/L)	3.0 [2.1-4.9]	3.0 [1.9-5.2]
Cum-lactate (min·mmol/L)	216 [0–2634]	138 [0–1245]
In-hospital mortality (%)	29	38

Data are presented as numbers and median [interquartile range]. SAPS-II, simplified acute physiology score; APACHE II, acute physiology, age and chronic health evaluation.

## Discussion

In the present study, lactate-time-integral was not superior in predicting in-hospital mortality compared with admission or maximum arterial lactate concentrations. Elevated admission lactate values, maximum lactate values during ICU stay, time-to-first-normalization, and lactate-time-integral were all associated with in-hospital mortality. Data on the first 24 hours after admittance revealed similar results: lactate derived variables were significantly higher in nonsurvivors compared with survivors. However, the “dynamic” lactate index, i.e., lactate-time-integral, did not outperform the “static” lactate variables, i.e., admission lactate and maximum lactate.

High lactate clearance within the first 6 hours has been shown to be associated with decreased 60-day mortality, even in the absence of arterial hypotension; survivors compared with nonsurvivors had a lactate clearance of 38 vs. 12%, respectively [14]. The present results also show a survival benefit for patients with lactate normalization within 6 hours in a heterogeneous ICU population. Recently, in a randomized study, the use of lactate clearance was described as an efficacious alternative for ScvO_2_-guided (target > 70%) resuscitation of patients in septic shock [19]. However, due to practical reasons, a treatment bias could not be excluded. Also, difference in protocol actions was small; despite a well-performed study, chance may have influenced the results. In the present study, we did not evaluate lactate clearance as described in septic patients by Nguyen et al. [14], i.e., as a ratio of lactate values. Instead, we evaluated a combination of the lactate-derived variables as a surrogate for lactate clearance. However, in the subgroup septic patients the lactate-time-integral or time-to-first-normalization did not outperform max-lactate. Lactime, described as a duration of hyperlactatemia, also has been shown to be the best discriminator of survival when the patients who died in the first 24 hours were excluded [10]. We performed a retrospective study and choose not to exclude those patients who died early after onset of the disease to picture the influence of lactate and lactate-derived variables in clinical reality. In conclusion, the present results recognize the importance of lactate clearance as described by others but also underline the importance of magnitude of lactate values during ICU treatment. In other words, time to first normalization may not be a key factor. This is clinically important: we need continuous reassessment of lactate levels to guide our resuscitation efforts.

A lactate threshold ≥4 mmol/L has been used to initiate protocol-based resuscitation [20,21]. Such an approach might imply acceptance of intermediate lactate levels in the range of 2 to 4 mmol/L. However, elevated mortality rates also are described in critically ill patients with only moderately elevated lactate levels during or even before admission to the emergency department (ED) [22-24]. Additionally, in two recent retrospective studies the relationship between lactate levels, lactate-derived variables, and outcome in critically ill patients was assessed. It was concluded that not only hyperlactatemia but also relative hyperlactatemia, i.e., lactate levels in the upper normal range, are associated with increased mortality [16,25]. Our results in ICU patients are concordant with these results, and we believe that normal values provide a reasonable clinical sign that tissue oxygenation is adequate and the metabolism is primarily aerobic. Of note, a higher lactate concentration threshold revealed different results: higher thresholds did not enhance predictive value for outcome. Indeed, not every hyperlactatemia is associated with acidosis, which is an important contributor to worse outcome. This might explain why lactate-time-integral does not outperform peak lactate values in outcome prediction in the present cohort. However, one could argue on the clinical relevance of the significant yet small difference in AUC_ROC_ of peak lactate and AUC_ROC_ of lactate-time-integral (0.692 vs. 0.676; *p* < 0.01). Definitive conclusions probably should not be made on such small differences. On the other hand, our results do not confirm superiority of dynamic lactate variables over static lactate variables as suggested earlier [16].

In addition, two other factors may be responsible for this finding. First, the retrospective design of our study and the lack of a specific intervention protocol limits the generalization of our results. Second, arterial lactate concentrations not only depend on lactate production but also on its clearance. It is not known whether one mechanism is more important than the other with respect to outcome prediction. Nevertheless, the mechanism causing hyperlactatemia may play an important role in outcome prediction, rather than the hyperlactatemia itself. For instance, as described in two recent reports [26,27], the severity of hyperlactatemia due to metformin accumulation alone does not predict outcome but even in those cases the causative role is uncertain. Also, comorbidities, such as renal insufficiency of liver failure, may play an additional role [26,27].

Finally, patients who died early, i.e., within 24 hours, possibly could not accumulate enough lactate-time-integral values despite disease severity. Establishment of a relationship between the lactate-time-integral and survival therefore could be disturbed. However, when the length of ICU stay was taken into account, the AUC_ROC_ was not larger and the lactate-time-integral per day did not perform better than peak lactate.

Nonsurvivors revealed shorter LOS_ICU_ and LOS_HOSP_, whereas lactate-time-integral was significantly higher in nonsurvivors compared with survivors. The duration and magnitude of increased lactate levels, represented by the area under the lactate curve, is associated with final outcome. Nevertheless, in the present study, the specificity and sensitivity, described by AUC_ROC_, of admission lactate and lactate-time-integral were similar in predicting in-hospital mortality. However, the present results on this large heterogeneous population underline the importance of ongoing watchfulness during the entire ICU stay.

Several limitations to our observations should be considered. First, this was a retrospective study, which precludes definitive conclusions. Also, there was no predefined lactate measurement or lactate-based goal-directed protocol. However, we consider the results strong enough to warrant further prospective studies analyzing the described phenomena, particularly because the data were collected over a 5-year period and derived from a large group of patients. Second, this was a single-unit study in one Dutch ICU, and therefore the results may only reflect the regional population and ICU management strategies. Nevertheless, we believe that selection bias was minimized, because all consecutive admissions were included in the data analysis and because there was no change in medical staffing, and admission and discharge criteria were stable during the study period. Third, we assumed a linear change in time between two lactate measurements, which is a simplification of a real biologic process. However, we believe that our approach represents an approximation with acceptable precision for the purpose of the present study.

## Conclusions

We conclude that hyperlactatemia is associated with in-hospital mortality in a heterogeneous ICU population. In our patients, lactate peak values predicted in-hospital mortality equally well as lactate-time-integral of arterial blood lactate levels above the upper normal threshold. In a heterogeneous ICU population, normalization of lactate within 6 hours is associated with lower mortality. Thus, concerning lactate levels in a heterogeneous ICU population magnitude matters and ongoing watchfulness on elevated lactate levels is warranted for time to first normalization may not be the key factor.

## Abbreviations

APACHE: Acute physiology age and chronic health evaluation; AUC_ROC_: Area under the ROC curve; DO_2_: Systemic oxygen supply; ED: Emergency department; ICU: Intensive care unit; LOS_ICU_: Length of ICU stay; LOS_HOSP_: Length of hospital stay; MODS: Multiple organ dysfunction syndrome; ROC: Receiver operating characteristic; RRT: Renal replacement therapy; SOFA: Sequential organ failure assessment; VO_2_: Systemic oxygen consumption.

## Competing interest

The authors state that they have no conflicts of interest.

## Authors’ contributions

PB drafted the manuscript and performed statistical analysis. LB participated in the design of the study and helped to draft the manuscript. SJ designed custom-made software. JR participated in the design of the study and helped to draft the manuscript. MK helped to draft the manuscript and provided general support. PS conceived of the study and participated in its design and coordination and helped to draft the manuscript. All authors have read and approved the final manuscript.
